# Collapsing Focal Segmental Glomerulosclerosis With Concurrent IgG4 Nephropathy

**DOI:** 10.7759/cureus.81031

**Published:** 2025-03-23

**Authors:** Eugene K Yeboah, Surya V Seshan, Fnu Pariya, Sulayman Khan, Muhammad Azhar, Moro Salifu, Subodh Saggi

**Affiliations:** 1 Internal Medicine, State University of New York Downstate Medical Center, Brooklyn, USA; 2 Pathology and Laboratory Medicine, Weil Cornell Medicine, New York, USA; 3 Nephrology, State University of New York Downstate Medical Center, Brooklyn, USA

**Keywords:** 1 collapsing fsgs superimposed with igg4, apol1 gene, collapsing fsgs, igg4 disease, igg4 nephropathy, igg4-related kidney disease, igg4 related tubulointerstitial nephritis

## Abstract

A 41-year-old male with a history of chronic kidney disease, hypertension, and psoriasis was referred to the nephrologist for worsening kidney function associated with nephrotic range proteinuria. The patient had no symptoms, but the initial workup showed elevated erythrocyte sedimentation rate (ESR), C-reactive protein (CRP), positive double-stranded DNA antibody(anti-DsDNA) but normal complement levels, normal antinuclear antibody (ANA) and negative beta-glycoprotein-1 IgG, IgM, and IgA. Further, the workup revealed the patient had elevated total immunoglobulin as well as elevated IgG subsets 2, 3, and 4. He was also found to have a high variant of apolipoprotein L1 (APOL1). A renal biopsy revealed diffuse active, subacute, and chronic interstitial inflammation, plasma cell-rich (25% IgG4 positive), confirming IgG4-related tubulointerstitial nephritis with concomitant IgG4 dominant, PLA2R negative membranous glomerulonephritis. There was also a severe podocytopathy in the form of diffuse segmental/global collapsing glomerulopathy with sclerosing changes as well as global glomerulosclerosis, extensive tubular atrophy with mild interstitial changes suggestive of a variant of focal segmental glomerulosclerosis (FSGS). A diagnosis of APOL-1 collapsing glomerulopathy with IgG4 nephropathy was made based on clinical and pathological findings. The patient’s kidney function stabilized, and IgG4 levels returned to normal after the patient was initiated on 60 mg daily prednisolone. The steroid was tapered off and the patient was started on mycophenolate mofetil 1000 mg twice daily. To our knowledge, this is the first reported case of IgG4-related kidney disease with concurrent severe APOL1-associated collapsing glomerulopathy.

## Introduction

IgG4-related disease (IgG4-RD) is a rare multisystemic autoimmune disorder characterized by interstitial infiltration of dominant IgG4-positive plasma cells and storiform fibrosis [1]. Tubulointerstitial nephritis (TIN) is the most common form of renal involvement in IgG4-related kidney disease [1]. Focal segmental glomerulosclerosis (FSGS) is a pathological lesion characterized by focal segmental sclerosis with effacement of foot processes [2]. FSGS can be categorized into primary, genetic, secondary, and FSGS of undetermined causes [2]. Genetic FSGS is associated with mutations in genes encoding proteins implicated in podocyte function or structure [2]. Positive high variant apolipoprotein L1 (APOL1) status confers higher odds for the development of genetic FSGS [2]. Podocytopathy associated with two high variant APOL1 alleles is associated with an earlier age of onset of FSGS and often leads to rapid progression to end-stage renal disease (ESRD) [3]. We present an unusual case of APOL-1 collapsing FSGS with concomitant IgG4 nephropathy.

## Case presentation

A 41-year-old male with a history of chronic kidney disease, hypertension, and psoriasis was referred to our renal clinic on May 17, 2024, for worsening kidney function associated with nephrotic range proteinuria. However, the patient had no complaints. The patient denied a history of smoking or alcohol intake. Apart from the patient’s sister who had a history of lymphoma, the patient did not have any significant personal or family history. The patient was compliant with his home medications, which included amlodipine 10 mg daily, atorvastatin 20 mg daily, eplerenone 25 mg daily, and ergocalciferol 1.25 mg daily. Physical examination was unremarkable except for class 1 obesity with a body mass index (BMI) of 32.3 kg/m^2^. The patient’s heart rate was 70 bpm and blood pressure was 151/86 mmHg. The patient's laboratory testing is summarized in Table 1

**Table 1:** Summary of patient's laboratory workup DNA: Deoxyribonucleic acid; HbA1C: glycated hemoglobin; IgG: Immunoglobulin; RPR: rapid plasma reagin; ANCA: antineutrophil cytoplasmic antibody; ESR: erythrocyte sedimentation rate; CRP: C-reactive protein; ANA: antinuclear antibody; APOL1: apolipoprotein L1

Parameter	Patient values	Reference range
Comprehensive metabolic panel		
Sodium	142 mmol/L	136-145 mmol/L
Potassium	4.4 mmol/L	3.5-5.1 mmol/L
Calcium	8.5 mg/dL	8.2-10.0 mg/dL
Chloride	104 mmol/L	98-107 mmol/L
Phosphorus	3.9 mg/dL	2.5-5 mg/dL
Uric acid	8.3 mg/dL	4.4-7.6 mg/dL
Creatinine	3.3 mg/dL	0.7-1.3 mg/dL
Blood urea nitrogen	49 mg/dL	7-25 mg/dL
Carbon dioxide	27 mmol/L	21-31 mmol/L
Glucose	94 mg/dL	70-99 mg/dL
Anion gap	10 mmol/L	10-20 mmol/L
Estimated glomerular filtration rate	23 mL/min/1.73m²	>60 mL/min/1.73m²
Liver function test		
Total bilirubin	0.3 mg/dL	0.3-1.0 mg/dL
Albumin	3.0 g/dL	3.5-5.7 g/dL
Total protein	6.4 g/dL	6.0-8.3 g/dL
Aspartate aminotransferase	13 U/L	13-39 U/L
Alanine aminotransferase	11 U/L	7-52 U/L
Alkaline phosphatase	70 U/L	34-104 U/L
HbA1C	5.1%	<5.7%
Complete blood count		
Hemoglobin	11.4 g/dL	14.0-18.0 g/dL
White blood count	6.78 k/μL	3.5-10.8 k/μL
Platelet	160 k/μL	130-400 k/μL
Hematocrit	35.9%	42.0-52.0%
Lipid panel		
Triglyceride	128 mg/dL	0-150 mg/dL
Total cholesterol	253 mg/dL	0-200 mg/dL
Low-density lipoprotein cholesterol	187 mg/dL	<99 mg/dL
High-density lipoprotein cholesterol	40 mg/dL	30-85 mg/dL
Anemia workup		
Iron	58 μg/dL	50-212 μg/dL
Total iron binding capacity	228 μg/dL	240-450 μg/dL
Unsaturated iron binding capacity	170 μg/dL	155-355 μg/dL
Ferritin	360.6 ng/mL	16.0-294.0 ng/mL
Urinalysis		
Appearance	Clear	Clear
pH	6.0	5.0-8.0
Specific gravity	1.013	1.005-1.030
Urine glucose	Negative	Negative
Urine Blood	Small	Negative
Urine creatinine	282 mg/dL	20-320 mg/dL
Urine protein	>1000	Negative
Urine nitrite	Negative	Negative
Leucocyte esterase	Negative	Negative
White blood cells (Urine)	<1/hpf	0-5/hpf
Urine cast	3/Ipf	0-2/Ipf
Urine protein creatinine ratio	5004 mg/g	0-200 mg/g
Infectious workup		
Human immunodeficiency virus 1/2 antigen/antibodies	Negative	Negative
Hepatitis C	Non-reactive	Non-reactive
Hepatitis B surface antigen	Non-reactive	Non-reactive
Tuberculosis quantiFERON gold	Negative	Negative
RPR	Negative	Negative
Glomerulopathy and autoimmune workup		
Complement (C3) levels	131 mg/dL	81-157 mg/dL
Complement (C4) levels	42 mg/dL	13-39 mg/dL
ESR	99 mm/hr	0-15 mm/hr
CRP	0.27 mg/dL	0.0-0.9 mg/dL
Beta-2 glycoprotein-1 IgG, IgM, and IgA	Negative	Negative
ANA	Negative	Negative
Anti-double stranded DNA	<49 IU/mL	<29 IU/mL
ANCA	Negative	Negative
Immunoglobulin G subset		
IgG subset 1	932 mg/dL	240-1118 mg/dL
IgG subset 2	552 mg/dL	123-549 mg/dL
IgG subset 3	165.6 mg/dL	15.0-102.0 mg/dL
IgG subset 4	262.5 mg/dL	1.0-123.0 mg/dL
Immunoglobulin G	1724 mg/dL	610-1660 mg/dL
Myeloma workup		
Monoclonal band	Negative	Negative
Kappa light chain	4.53 mg/dL	3.3-19.4 mg/dL
Lambda light chains	12.2 mg/dL	5.5-26.3 mg/dL
Genetic test		
APOL1 high variant	Positive	Negative

Imaging

**Figure 1:**
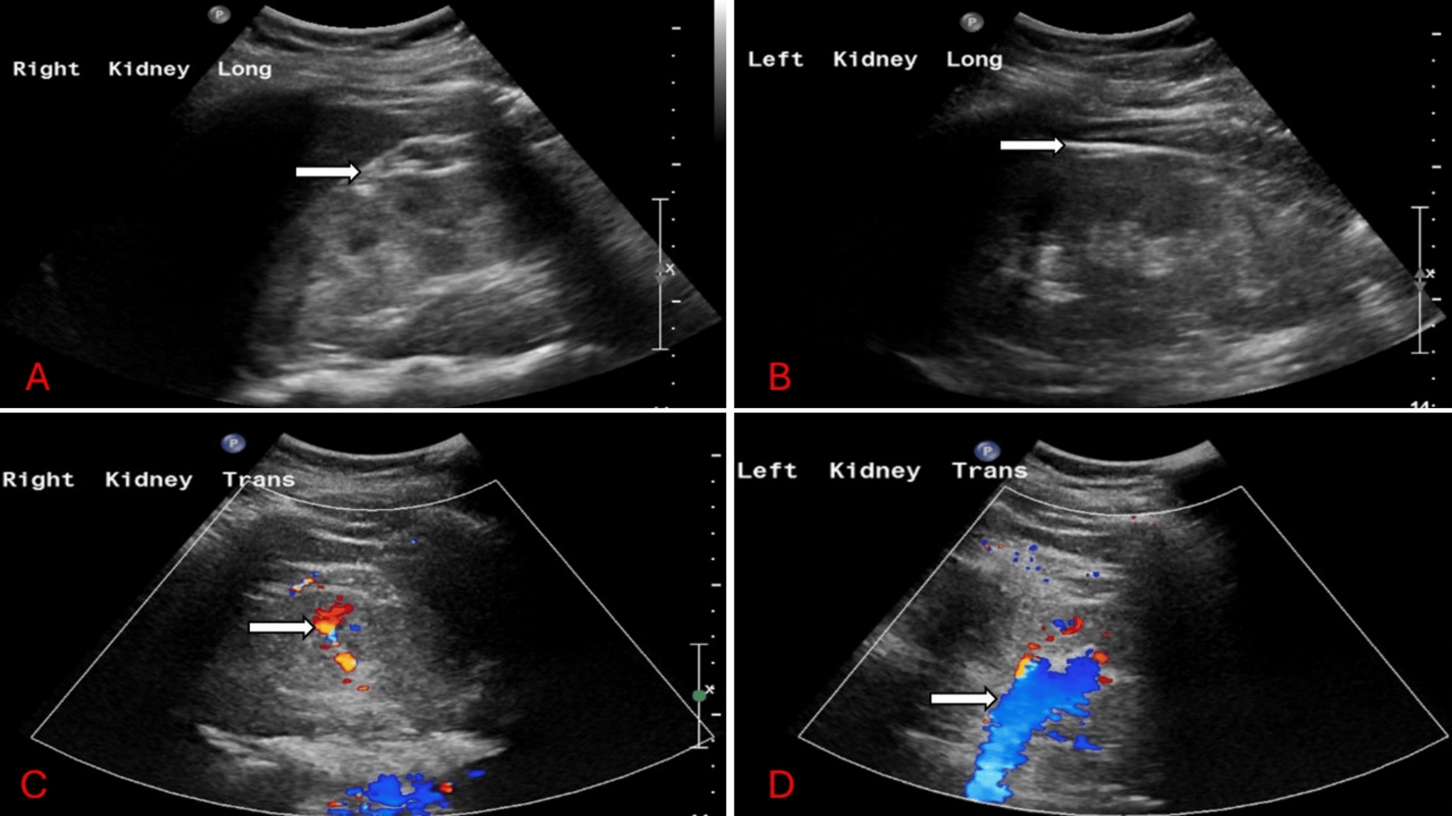
Ultrasound of patient's kidneys A: The right kidney measures 11.5 cm x 6.1 cm x 5.9 cm with increased echogenicity (arrow) but normal parenchyma thickness and contour and no pelvicalyceal dilatation, calculi, cysts, or solid masses. B: The left kidney measures 12.1 cm x 6.1 cm x 6.3 cm with increased echogenicity with corticomedullary differentiation (arrow) but normal parenchyma thickness and contour and no pelvicalyceal dilatation, calculi, cysts, or solid masses. C: Doppler ultrasound (arrow) of the right kidney. D: Doppler ultrasound (arrow) of the left kidney.

Figure 1 shows the patient's kidney ultrasound. It showed increased echogenicity in both kidneys but normal parenchyma thickness and contour and no pelvicalyceal dilatation, calculi, cysts, or solid masses. Echogenic kidneys are consistent with medical renal disease.

Kidney biopsy

**Figure 2:**
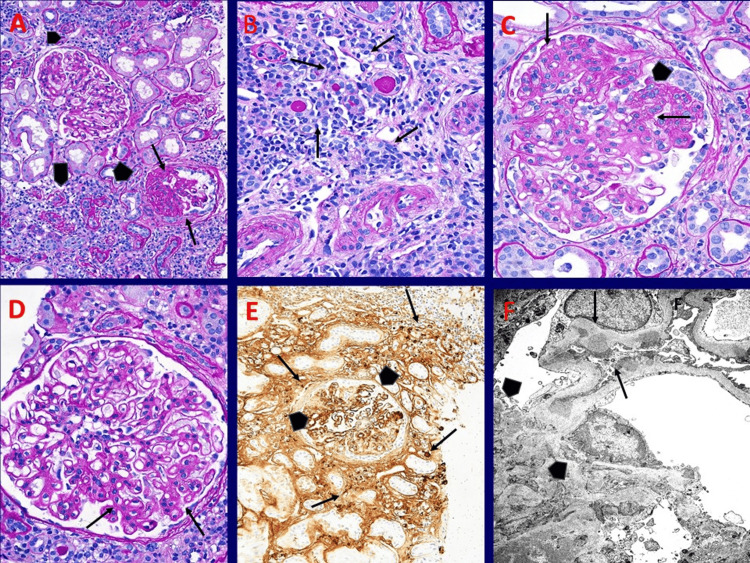
Kidney biopsy of patient A: Renal cortical tissue with two glomeruli, one showing segmental to partial collapse with sclerosing changes (arrows), while the other shows mild irregular thickening of the capillary walls, along with areas of active and chronic interstitial inflammation (arrowheads) surrounding atrophic tubules. PAS stain X200 B: High magnification of interstitial inflammation, composed mainly of plasma cells (arrows), admixed with some lymphocytes and occasional eosinophils, with preserved arterial vessels. PAS stain X400 C: Glomerulus showing segmental collapsing features (arrows) with early sclerosing changes, covered by hyperplastic epithelial cells (arrowhead). PAS stain X400 D: Glomerulus showing moderate to marked irregular thickening of the peripheral capillary walls with early spikes on the outer aspect (arrows), along with focal mild mesangial prominence. PAS stain X400 E: Immunoperoxidase stain on paraffin-embedded tissue using a polyclonal antibody to IgG4 shows increased positive staining within infiltrating plasma cells (arrows), as well as granular IgG4 staining of the glomerular capillary walls (arrowheads) in deposits on a segmentally sclerosed glomerulus. IHC for IgG4 X200 F: Electron microscopy of a portion of glomerulus showing subepithelial electron-dense deposits of varying sizes with early basement membrane spikes (arrows) as well as mesangial deposits (arrowheads). X5000 PAS: periodic acid-schiff; IHC: Immunohistochemistry

Figure 2 shows that the patient's renal biopsy was performed on July 10, 2024, and processed routinely for light microscopy, immunofluorescence, and electron microscopy. The tissue showed diffuse active, subacute, and moderate to severe chronic interstitial inflammation, with dominant IgG4 positive, plasma cell-rich, diagnostic of IgG4-related TIN along with a secondary form of membranous glomerulonephritis with IgG4 dominant deposits that are negative for phospholipase A2 receptor (PLA2R), neural epidermal growth factor-like 1 (NELL1), thrombospondin 7A and exostosin I&II using immunohistochemical techniques. There was also diffuse segmental/global collapsing glomerulopathy with sclerosing changes as well as global glomerulosclerosis, extensive tubular atrophy with mild interstitial fibrosis.

The patient was started on prednisolone 60 mg daily and tapered, based on the renal biopsy histological findings of APOL-1 collapsing FSGS along with IgG4-related tubulo-interstitial nephritis and membranous glomerulonephritis with dominant IgG4 deposits. Whilst the steroid was tapered off, he was started on mycophenolate mofetil 1000 mg twice daily. Education about kidney transplants and early referral started.

**Figure 3:**
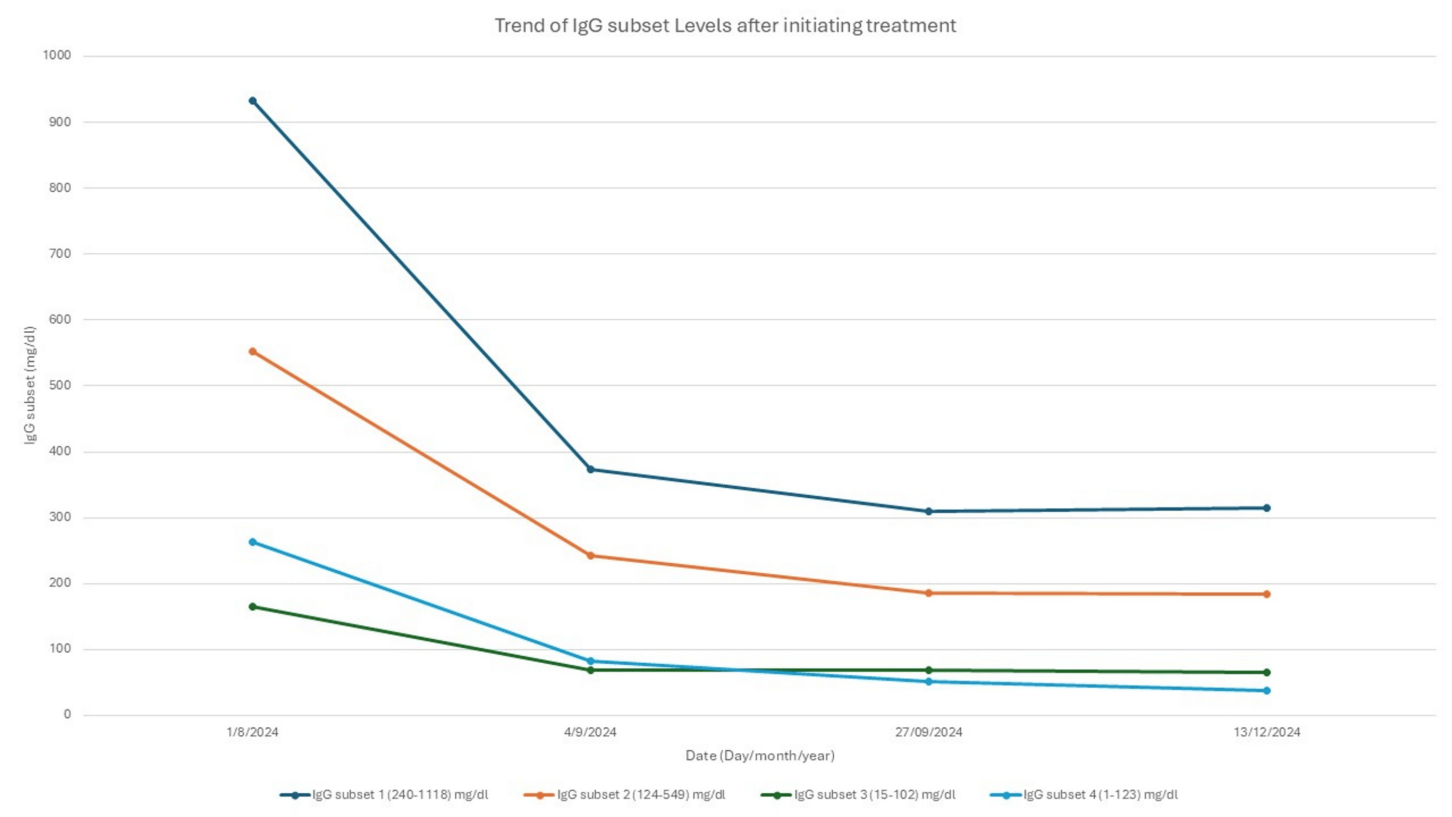
Trend of immunoglobulin subsets after initiating treatment IgG subset 1 reference range (240-1118) mg/dL. IgG subset 2 reference range (124-549) mg/dL. IgG subset 3 reference range (15-102) mg/dL. IgG subset 4 reference range (1-123) mg/dL. IgG: immunoglobulin

Figure 3 shows the trend of IgG4 subset levels after initiating treatment. 

## Discussion

IgG4-RD is a group of multisystemic chronic relapsing-remitting autoimmune diseases, characterized by tissue infiltration with lymphocytes and IgG4-secreting plasma cells leading to fibrosis [4]. The most commonly affected organs are the pancreas, biliary tree, salivary and lacrimal glands, retroperitoneum, and lymph nodes. IgG4-RD was initially recognized in the pancreas as a cause of autoimmune pancreatitis; however, the distinctive features of IgG4-RD have been described in nearly every organ system, including the kidneys [5]. A study in 2023 estimated the overall incidence of ІgG4-RD to be 0.78 to 1.39 per 100000 person-years [6]. FSGS is a form of glomerular podocytopathy presenting with a clinicopathologic syndrome of proteinuria, usually of nephrotic range, associated with focal and segmental sclerotic glomerular lesions, some showing collapsing features with epithelial hyperplasia [7]. FSGS is a common histopathologic lesion among adults with nephrotic syndrome in the United States, accounting for 35% of all cases [8]. FЅGS is also the most common primary glomerular disease identified in patients with end-stage kidney disease (ESKD) in the United States [9].

IgG4-RD has variable clinical presentation, and diagnosis relies on extensive workup including clinical, serological, radiological, and histological assessment [10]. A thorough history and clinical examination may reveal previously unidentified features of IgG4-RD [10,11]. Workup may reveal an elevated total serum eosinophil, IgE, IgG4, total protein, and low complement levels [12]. The predominant features associated with IgG4-RD on kidney biopsies are plasma cell-rich TIN with increased IgG4-positive plasma cells and fibrosis [12]. Some glomerular pathologies have been reported to be concurrent with IgG4-TIN, although TIN is the most common lesion in IgG4-nephropathy [12]. Membranous nephropathy is the most common glomerular lesion with dominant IgG4 deposits [13,14]. To our knowledge, there are no recorded cases of IgG4-related kidney disease concurrent with a severe podocytopathy associated with APOL1 gene variants leading to segmental and global collapsing glomerulopathy with sclerosing changes.

FЅGS is classified based on clinical presentation and pathologic lesions on kidney biopsy [2]. The classification includes primary FSGS, secondary FSGS, genetic FSGS, and FSGS of undetermined causes [2]. Differentiating these classes of FSGS is imperative as it has both therapeutic and prognostic implications. Primary FSGS is associated with the presence of an unknown circulating factor, with no evidence of other underlying etiology [15]. Although Primary FSGS is common in adolescents and young adults, it may occur at any age and usually presents as nephrotic-range proteinuria, hypoalbuminemia, and hyperlipidemia [16]. Secondary FSGS, in contrast, arises from various systemic conditions or external factors affecting the kidney and typically leads to non-nephrotic range proteinuria and normal albumin levels [16]. Genetic FSGS can present early in childhood with nephrotic syndrome, which is often steroid-resistant, or in some forms in adulthood with less severe proteinuria. Mutations in genes encoding proteins involved in the slit diaphragm, cell membrane, cytoskeleton, nuclear, mitochondrial, and lysosomal functions of the kidneys have been implicated in genetic FSGS [17]. Additionally, polymorphisms in APOL1, commonly found in individuals of African descent, significantly increase the risk of developing genetic FSGS [18].

All patients with IgG4-RD who have symptoms or active disease require treatment to prevent irreversible complications [10]. Glucocorticoids are the first-line treatment in IgG4-RD unless contraindicated [10]. The rate of relapse is high, hence, maintenance therapy with low-dose steroids or a steroid-sparing agent, such as rituximab, azathioprine, mycophenolate mofetil, or methotrexate, may be necessary for patients where the risk of disease recurrence is high, or steroids cannot be tapered due to persistent disease [10]. Steroids are a cornerstone in the management of primary FSGS [18]. For patients who are steroid-resistant or experience adverse effects from steroids, alternative immunosuppressants such as calcineurin inhibitors such as tacrolimus and cyclosporin [18]. Management of secondary FSGS primarily focuses on treating the underlying condition, underscoring the importance of comprehensive serological evaluation and identifying secondary etiologies [18]. Patients with genetic etiology are more likely to have steroid- and immunosuppressant-resistant disease [18]. However, these patients rarely experience recurrent disease in a transplanted kidney, making them good transplant candidates [18].

## Conclusions

FSGS and IgG4-related kidney disease are two distinct disease entities. There have been reported cases of IgG4-related kidney disease with concurrent glomerular diseases, such as membranous nephropathy, but this is the first reported case of IgG4-related kidney disease with concurrent FSGS associated with an APOL1 gene variant. Most cases of both conditions respond well to steroids and other immunosuppressants.
